# Detection of the electromechanical delay and its components during voluntary isometric contraction of the quadriceps femoris muscle

**DOI:** 10.3389/fphys.2014.00494

**Published:** 2014-12-23

**Authors:** Haris Begovic, Guang-Quan Zhou, Tianjie Li, Yi Wang, Yong-Ping Zheng

**Affiliations:** Interdisciplinary Division of Biomedical Engineering, Faculty of Engineering, The Hong Kong Polytechnic UniversityKowloon, China

**Keywords:** electromechanical delay, excitation-contraction coupling, series elastic components, contractile components, rectus femoris muscle, transient ultrasound

## Abstract

Electromechanical delay (EMD) was described as a time elapsed between first trigger and force output. Various results have been reported based on the measurement method with observed inconsistent results when the trigger is elicited by voluntary contraction. However, mechanomyographic (MMG) sensor placed far away on the skin from the contracting muscle was used to detect muscle fiber motion and excitation-contraction (EC) coupling which may give unreliable results. On this basis, the purpose of this study was to detect EMD during active muscle contraction whilst introducing an ultrafast ultrasound (US) method to detect muscle fiber motion from a certain depth of the muscle. Time delays between onsets of EMG-MMG, EMG-US, MMG-FORCE, US-FORCE, and EMG-FORCE were calculated as 20.5 ± 4.73, 28.63 ± 6.31, 19.21 ± 6.79, 30.52 ± 8.85, and 49.73 ± 6.99 ms, respectively. Intrarater correlation coefficient (ICC) was higher than MMG when ultrafast US was used for detecton of the Δt EMG-US and Δt US-FORCE, ICC values of 0.75 and 0.70, respectively. Synchronization of the ultrafast ultrasound with EMG and FORCE sensors can reveal reliable and clinically useful results related to the EMD and its components when muscle is voluntarily contracted. With ultrafast US, we detect onset from the certain depth of the muscle excluding the tissues above the muscle acting as a low-pass filter which can lead to inaccurate time detection about the onset of the contracting muscle fibers. With this non-invasive technique, understanding of the muscle dynamics can be facilitated.

## Introduction

Electromechanical delay (EMD) was described as a time elapse between the onset of muscle electrical activation and onset of force production, reflecting both electrochemical processes [i.e., synaptic transmission, propagation of the action potential, excitation-contraction (EC) coupling] and mechanical processes [i.e., force transmission along the active and passive parts of the series elastic components (SECs)] (Cavanagh and Komi, 1979; Esposito et al., 2009, 2011a,b; Hug et al., 2011a,b). Different results had been reported such as 8.5 ms during supramaximally stimulated tibial nerve and 125 ms during voluntary elicited contractions (Blackburn et al., 2009; Yavuz et al., 2010). Following the trigger for contraction, the contractile components (CCs) firstly stretch the SECs before the force output is evident (Cavanagh and Komi, 1979; Bell and Jacobs, 1986; Muraoka et al., 2004). When the trigger is electrical stimulation, cortical inputs are bypassed (Shultz and Perrin, 1999), stimulus cross-talk effect is minimized (Sasaki et al., 2011) and force output occurs at significantly shorter time than the voluntary initiated contraction. During voluntary muscle contraction, cortical input is required for voluntary motor control and responses occur at a significantly greater delay (Zhou et al., 1995; Shultz and Perrin, 1999; Hopkins et al., 2007).

Recently, time delay between EMG signal and FORCE output (EMD) elicited by electrical stimulation, was partitioned into the time delays between the EMG and MMG (time index of local sarcomere motion prior to the elongation of the passive series elastic components) and MMG and Force (monitor of the overall events after cross-bridge formation) (Esposito et al., 2011a). Physiologically, the time delay between EMG-MMG could describe Ca^2+^ release and sensitivity, its involvement during the EC coupling and association between dihydropyridine and ryanodine receptors (Esposito et al., 2011a). For detecting the onset of the fiber motion, MMG signal was used and attributed to the dimensional changes of the active muscle fibers (Herda et al., 2010; Esposito et al., 2011a,b; Sasaki et al., 2011; Camic et al., 2013). Using MMG, it was found as 2.2 ± 0.3 ms in gastrocnemius muscle (Esposito et al., 2011a) and lower than 5 ms in biceps muscle (Sasaki et al., 2011) when the trigger was initiated by electrostimulation. Whether MMG is an appropriate method for detection of the fiber activation remains unclear because of its far away placement from the real contracting muscle. Even more, there are reported cross-talk from the adjacent muscles when MMG sensor was used (Beck et al., 2010). In some studies, new way of imaging the motion of an *in vivo* contractile muscle was introduced by using an ultrafast US scanner (Deffieux et al., 2006, 2008; Nordez et al., 2009; Lacourpaille et al., 2013). Using ultrafast US with rate of 4 kHz, the onset of the fiber activation was detected as 6.05 ± 0.64 ms from gastrocnemius muscle (Nordez et al., 2009) and 3.9 ± 0.2 ms from the biceps muscle (Lacourpaille et al., 2013) in reference to the given time of electrostimulation. Time delay after fiber activation to the force output, corresponding to the changes in the tendon was found as 11.65 ± 1.27 ms in the gastrocnemius (Nordez et al., 2009) and 11.8 ± 2.2 ms in the biceps muscle (Lacourpaille et al., 2013). Despite these findings, it still remains unknown, how much these time delays corresponding to the EC coupling and force transmission along the SEC coincide with the structural changes displayed by electrophysiological signals during voluntary muscle contraction. To our knowledge, partitioning of the EMD (EMG-FORCE) into the time delays corresponding to the time between EMG onset and onset of the fiber motion and onset of the actual force production have not been investigated using both MMG and ultrafast US during *voluntary muscle contraction*. We hypothesis that using ultrafast US and tracking the onset of the fiber motion from a certain depth can reveal more reliable results regarding the time delays corresponding to the muscle fiber activation and actual force production. MMG sensor may be disadvantageous because of its far away placement from the contracting muscle and interspaced non-contractile tissues between muscle and MMG sensor.

On this basis, we designed the present study to find out EMD and time delays corresponding to the EC coupling and SEC during active muscle contractions when both MMG and ultrafast US were used for detection of the muscle fiber activation.

## Materials and methods

### Participants

A group of 14 men, young volunteers were recruited in the study. They were all healthy subjects without any history of previous injury, metabolic or neurologic disease. The physical and anthropometric characteristics of the participants are given in the Table 1. No one of them were involved in any vigorous exercise on a daily basis. The human subject ethical approval was obtained from the relevant committee in the Hong Kong Polytechnic University and informed consent was obtained from each subject prior to the experiment.

**Table 1:** **Physical and anthropometric characteristics of the participants (*n* = 14; mean ± SD)**.

Age (years)	28.2 ± 3.25
Weight (kg)	71.8 ± 10.16
Height (cm)	172.4 ± 5.84
BMI	24.1 ± 2.62
Mid-sagittal thickness of the RF (mm)	20.4 ± 2.40

#### Experimental protocol

**Figure 1:**
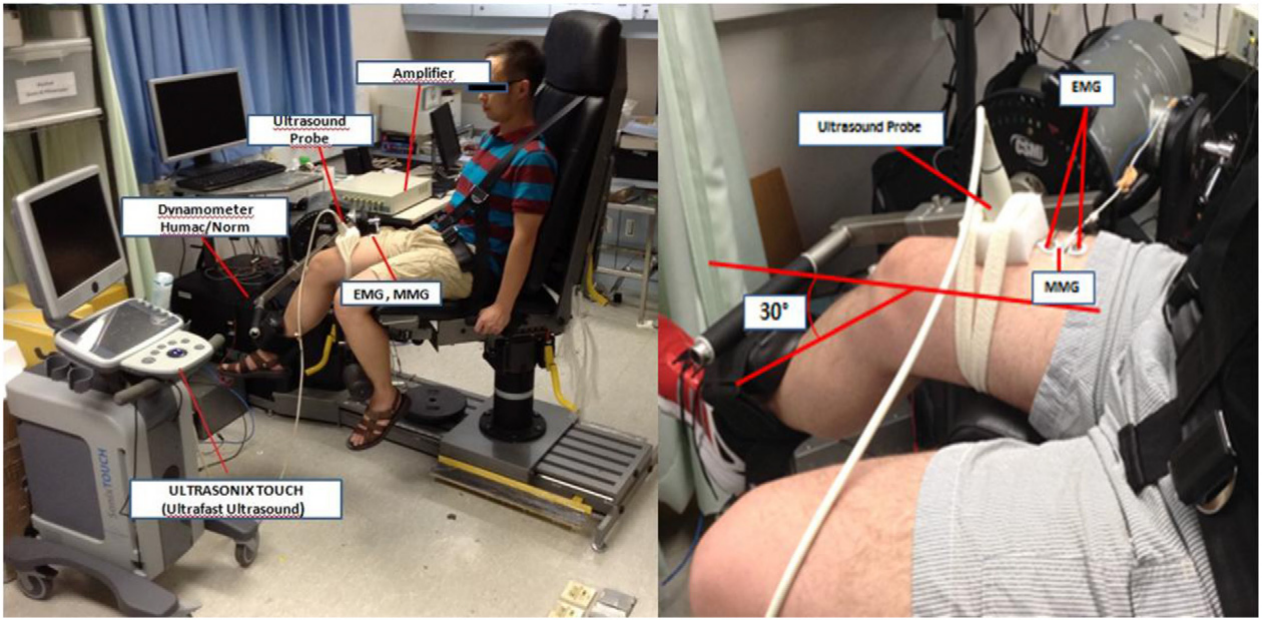
**Experimental setup for the measurement of the surface EMG, MMG, FORCE, and US onsets from the right (dominant) rectus femoris muscle during isometric contraction of the quadriceps femoris muscle.** The subject is seated with the back inclination of 80° and right knee was adjusted at flexion angle of 30° below the horizontal plane on a calibrated dynamometer (Humac/Norm Testing and Rehabilitation System, Computer Sports Medicine, Inc., MA, USA).

The dominant leg for being tested was defined as a leg with which the subject preferred to kick a ball. After the anthropometric measurements, subject was seated with a back inclination of 80° and knee was adjusted at flexion angle of 30° below the horizontal plane on a calibrated dynamometer (Humac/Norm Testing and Rehabilitation System, Computer Sports Medicine, Inc., MA, USA). Straps across the subject's trunk were used to stabilize hip and trunk movement (Figure 1). The 30° was chosen to activate the muscle with minimum pre-stretching of the muscle fibers because increased slack within the muscle-tendon unit (MTU) produced by increasing flexion angles may affect the shortening velocity of the fastest muscle fibers and consequently effect results (Sasaki et al., 2011).

**Figure 2:**
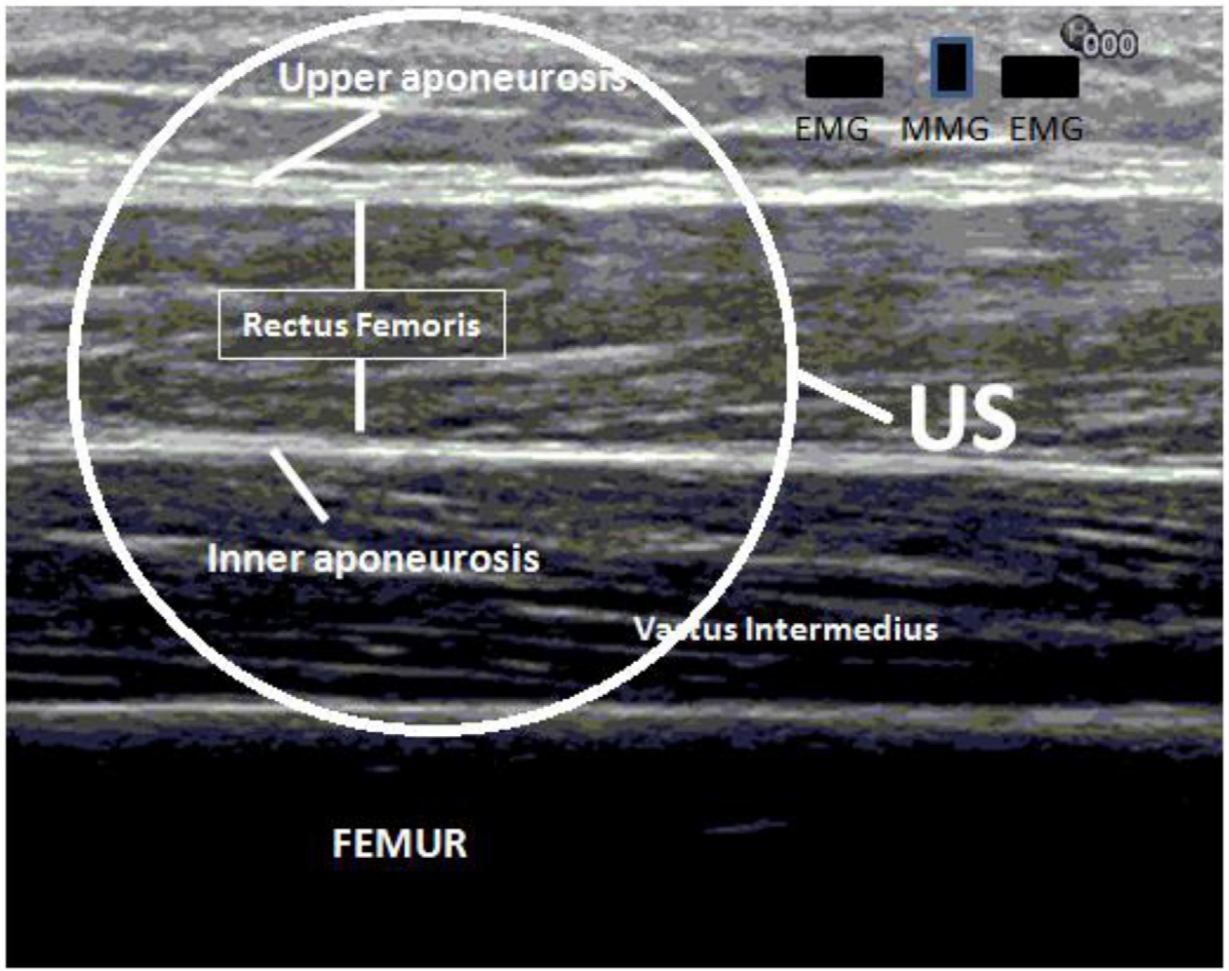
**The aponeuroses appears as hyperechoic strips and the distance between upper and inner aponeuroses is used for measuring the thickness and depth of the rectus femoris muscle.** Measured real depth is then used for the calculation of the US onset during isometric contraction of the quadriceps femoris muscle.

The rectus femoris muscle (RF) was chosen for testing because of its surface position. The thickness of the RF muscle was first measured using a commercial ultrasound scanner (Ultrasound Diagnostic Scanner, EUB-8500, Hitachi Medical Corporation, Tokyo, Japan) with a 7.5 MHz linear array ultrasound probe. For the thickness measurement, the ultrasonographic image was obtained at approximately 60–70% of the tight length from the popliteal crease to the greater trochanter corresponding to the muscle belly of the RF (Ryoichi et al., 2013). The thickness of the RF was measured as a distance between upper and inner aponeuroses. Measured thickness of the RF muscle was summed with the thickness of the skin and fat layer and used as a predefined region for the following US A-mode signal analysis (Figure 2).

The experimental procedure was explained in details and familiarization session was given to allow the subject to practice the isometric contractions at very low load just a few times without producing muscle fatigue. Muscle activity during voluntary isometric contractions was recorded simultaneously by EMG, MMG, Force and ultrafast US while the subject was seated on the calibrated dynamometer (Humac/Norm Testing and Rehabilitation System, Computer Sports Medicine, Inc., MA, USA) with knee flexion angle adjusted at 30°. The test procedure consisted of 4 isometric contractions of the Quadriceps Femoris (QF) muscle with resting period of 2 min between contractions to prevent muscle fatigue. During the test, the subject was asked to apply maximum isometric contraction as quickly as possible in 1 s and to keep it approximately 3 s. Verbal order was given to the subject about the start and termination of the muscle contraction. The order “start” was given immediately after starting the collection of A-mode signals in the ultrafast US device. After the termination of each contraction, the position of the US probe was checked to ensure that there was not any displacement of the probe caused by the movement artifact of the muscle during contraction.

#### Data acquisition

Two surface EMG bipolar Ag-AgCl electrodes (Axon System, Inc., NY, USA) for differential EMG detection were placed on the RF muscle belly, approximately at the 50–60% of the distance between the spina iliaca anterior superior and superior patellar margin. To reduce the skin impedance, skin was cleaned with isopropyl alcohol and abraded with fine sandpaper. The ground electrode was placed over the tibial crest. For detecting the MMG signal, a monodirectional accelerometer (EGAS-FS-10-/V05, Measurement Specialties, Inc., France) was fixed between two surface EMG electrodes. Together with the accelerometer, interelectrode distance between two surface EMG electrodes was 30 mm. The surface EMG and MMG signals were amplified by a custom-designed amplifier with a gain of 2000, filtered separately by 10–1000 Hz and 5–1000 Hz bandpass analog filters within the amplifier, respectively. The isometric force generated by the quadriceps femoris muscle was measured using a dynamometer (Humac/Norm Testing and Rehabilitation System, Computer Sports Medicine, Inc., MA, USA). The EMG, MMG, and FORCE signals were digitized with a sampling rate of 4 KHz, and stored on a personal computer when the subject performed voluntary isometric contraction.

**Figure 3:**
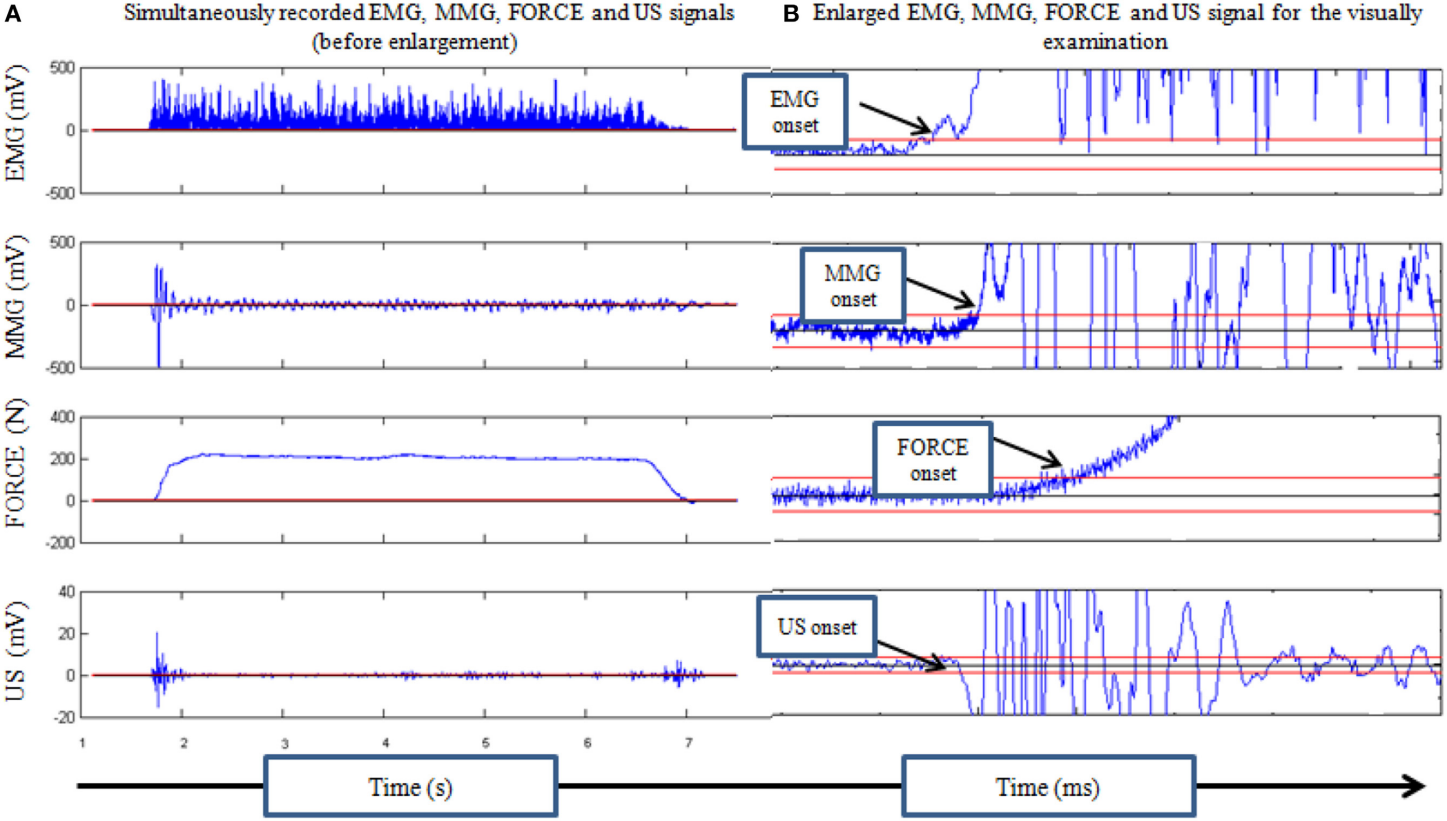
**(A)** Presentation of the rectified EMG signal, MMG, US, and FORCE signals simultaneously recorded during isometric contraction of the quadriceps femoris muscle. **(B)** Signals were calculated offline using designed program in MatLab and visually examined. Calculated delays between each onset were expressed in milliseconds.

A commercial ultrasound scanner (Sonix Touch, Analogic Corporation, USA) with a 7.5 MHz linear array ultrasound probe (Ultrasonix L14-5/35) was used to collect the ultrasound A-mode signal, which could reach a very high frame rate. The US recording was made by a custom program installed in a programmable ultrasound scanner (Ultrasonix Touch, Analogic Corporation, Massachusetts, USA) to achieve a very high frame ultrasound scanning at a selected location. The US probe was placed as close as possible to the surface EMG electrodes in longitudinal direction along the muscle fibers of the RF muscle. Ultrasound gel was applied between the skin and probe to serve as an acoustic coupling medium. To avoid probe motion artifact which may cause misleading of the real onset (Vasseljen et al., 2006), US probe was fixed in a foam holder and bandage was used without unacceptable tightening to prevent sliding of the probe during contraction. After the placement and fixation of the ultrasound probe, B-mode image was checked to ensure that US probe was on the RF muscle. The whole data acquisition procedure started with the collection of EMG, MMG, and FORCE signals. Then the collection of A-mode US signals was started, and the verbal instruction of “start” was given by the operator. The A-mode US signal was collected at a frame rate of 4 k frames/s for 10 s during the voluntary isometric contraction. After the first frame of A-mode signal was collected, a signal was generated by the ultasound scanner and outputted as an external trigger signal, which was inputted into the device for EMG/MMG/FORCE signal collection. This channel of trigger signal was used for synchronizing the collection of A-mode US signal with other signals. The recorded US signal was processed to detect the root mean square (RMS) value of the selected region of interest (ROI) (Figure 2). This RMS value obtained from each frame of US signal was then substracted by the RMS value of the first frame, and the result was used to form new signal representing the US signal disturbance induced by the muscle contraction (Figure 3A).

### Data analysis

Collected signals were processed off-line using a program written in MatLab (version 2008a, USA). Time delays between EMG and MMG (Δ*t* EMG-MMG), MMG and FORCE (Δ*t* MMG-FORCE), EMG and US (Δ*t* EMG-US), US and FORCE (Δ*t* US-FORCE) and EMG and FORCE (Δ*t* EMG-FORCE) onsets were calculated off-line. EMG signal was rectified and condition of three standard deviations (SDs) from the mean baseline noise was observed for detecting the onset of each signal. In order to define a crossing time as the onset time, a condition for signal to stay 10 ms above the threshold level was set by the program and visually examined. The time delays, Δ*t* EMG-MMG, Δ*t* MMG-FORCE, Δ*t* EMG-US, Δ*t* US-FORCE, and Δ*t* EMG-FORCE were calculated for each contraction and expressed in milliseconds (Figure 3B).

### Statistical analysis

The data were analyzed with a software package SPSS V.19 (IBM SPSS Statistics for Windows, Version 19.0. Armonk, NY, USA). The normal distribution of the data was analyzed by the Kolmogorov-Smirnov test. Values are reported as means ± SD. To check whether there are any differences between the contractions, One-Way analysis of variance (ANOVA) for repeated measures was used. To determine the repeatability of all our measurements, the standard error in measurement (SE) and intraclass correlation coefficient ICC (2,k) were calculated using the means calculated between onsets of the 4 voluntary contractions to express agreement between contractions. To test the differences between Δt EMG-MMG − Δt EMG-US and Δt MMG-FORCE − Δt US-FORCE, paired *t*-test was used. The statistical significance was set at the 0.05 level. Percentage of each time delay relative to the overall time delay (EMD) was also reported.

## Results

The results about the demographic characteristics of the subjects are demonstrated in the Table 1. To check the normal distribution of the data, Kolmogorov-Smirnov test revealed that Δt EMG-MMG, Δt EMG-US, Δt US-FORCE, and Δt EMG-FORCE are normally distributed while Δt MMG-FORCE was non-normally distributed (*P* < 0.05). As no differences were observed between 4 voluntary isometric contractions using One-Way ANOVA for repeated measures (Δt EMG-MMG; *P* > 0.05, Δt MMG-FORCE; *P* > 0.05, Δt EMG-US; *P* > 0.05, Δt US-FORCE; *P* > 0.05 and Δt EMG-FORCE; *P* > 0.05) average and SDs were calculated for each time delay.

The averages ± SD for the Δt EMG-MMG, Δt MMG-FORCE, Δt EMG-US, Δt US-FORCE, and Δt EMG-FORCE were calculated as 20.5 ± 4.73 ms, 28.63 ± 6.31 ms, 19.21 ± 6.79 ms, 30.52 ± 8.85 ms, and 49.73 ± 6.99 ms, respectively (Table 3). The relative contribution of the Δt EMG-MMG, Δt MMG-FORCE, Δt EMG-US, and Δt US-FORCE to the overall time delay (Δt EMG-FORCE, EMD considered as 100%) were expressed in percentage and found as 41.1%, 57.1%, 38.9%, and 60.92%, respectively (Table 3). To compare two different methods for detection of the muscle fiber activation onset, paired *t*-test did not reveal any significant difference between Δt EMG-MMG and Δt EMG-US (20.5 ± 4.73; 19.21 ± 6.79, *p* > 0.05) and Δt MMG-FORCE and Δt US-FORCE (28.63 ± 6.31; 30.52 ± 8.85, *p* > 0.05). Significant differences were found between Δt EMG-MMG and Δt MMG-FORCE (*p* < 0.05) and Δt EMG-US and Δt US-FORCE (*p* < 0.05).

**Table 2:** **Repeatability (ICC, 2*k*) between 4 isometric contractions of the quadriceps femoris muscle**.

**ICC between 4 repeated contractions**			
**Time delays (Δt) between onsets**	**95% confidence interval**		
	**ICC, 2*k***	**Lower bound**	**Upper bound**
Δt EMG-MMG	0.488	−0.156	0.816
Δt MMG-FORCE	0.570	0.03	0.846
Δt EMG-US	0.751	0.437	0.910
Δt US-FORCE	0.707	0.338	0.895
Δt EMG-FORCE (EMD)	0.632	0.169	0.868

**Table 3:** **Averages ± SD of the four contractions, percentage (%EMD) and standard error in measurement (SEM) for each time delay; Δt EMG-MMG, Δt MMG-FORCE, Δt EMG-US, Δt US-FORCE, and Δt EMG-FORCE (EMD)**.

**Time delays between EMG and MMG, MMG and FORCE, EMG and US, US and FORCE and EMG and FORCE (EMD) for each subject**									
**Subjects**	**Δt EMG-MMG (ms)**	**%EMD**	**Δt MMG-FORCE (ms)**	**%EMD**	**Δt EMG-US (ms)**	**%EMD**	**Δt US-FORCE (ms)**	**%EMD**	**Δt EMG-FORCE, EMD (ms)**
1	17.9 ± 5.18	43.20	23.5 ± 1.20	56.70	24.92 ± 6.83	60.10	16.47 ± 3.09	39.79	41.4 ± 4.86
2	15 ± 5.79	33.97	29.15 ± 4.21	65.90	12.87 ± 4.77	28.90	31.27 ± 6.34	70.831	44.15 ± 6.11
3	29.92 ± 7.42	53.60	23.92 ± 3.87	42.90	24.55 ± 4.06	43.90	31.15 ± 7.81	55.92	55.7 ± 8.07
4	18.25 ± 7.35	33	36.87 ± 826	66.70	21.9 ± 5.48	39.70	33.22 ± 7.67	60.27	55.12 ± 4.96
5	16.85 ± 5.19	48.50	12.3 ± 10.79	35.44	14.47 ± 4.27	41.40	20.22 ± 10.78	58.28	34.7 ± 10.97
6	15.15 ± 4.15	33.30	30.25 ± 1.92	66.50	20.07 ± 13.28	44	25.32 ± 12.18	56	45.4 ± 16.5
7	16.45 ± 6.54	33.70	32.32 ± 1.92	66.30	13.0 ± 9.33	26.60	35.77 ± 7.39	73.34	48.7 ± 5.21
8	17.72 ± 10.96	35.10	32.7 ± 11.82	64.80	7.7 ± 3.7	15.20	42.72 ± 14.16	84.72	50.42 ± 12.31
9	26 ± 8.76	46.20	30.2 ± 4.28	53.70	19.95 ± 7.75	35.40	36.25 ± 12.55	64.50	56.2 ± 5.24
10	20.27 ± 8.58	36.50	34.95 ± 15.14	63.20	27.57 ± 6.24	49.80	27.65 ± 7.93	50.06	55.22 ± 8.09
11	27.1 ± 5.33	43.90	34.5 ± 5.81	56	18.45 ± 1.21	29.80	43.15 ± 8.66	70.04	61.6 ± 7.86
12	20.55 ± 4.15	43.80	26.37 ± 4.37	56.10	31.97 ± 0.97	68.10	14.82 ± 6.73	31.67	46.8 ± 5.84
13	25.1 ± 8.36	50.80	24.3 ± 4.67	49.10	11.3 ± 8.4	22.80	38.1 ± 10.17	77.12	49.4 ± 4.81
14	20.8 ± 2.78	40.40	29.47 ± 4.50	57.10	20.25 ± 10.23	39.20	31.15 ± 10.78	60.60	51.4 ± 4.31
Mean	20.5	41.10	28.63	57.10	19.21	38.90	30.52	60.92	49.73
SD	4.73	7	6.31	9.50	6.79	14.20	8.85	14.29	6.99
SEM	0.9		1.18		1.15		1.59		1.3

The SE values calculated for the Δt EMG-MMG, Δt MMG-FORCE, Δt EMG-US, Δt US-FORCE, and Δt EMG-FORCE were 0.9, 1.18, 1.15, 1.59, and 1.3 ms, respectively. However, between 4 contractions, intraclass correlation coefficient (ICC, 2*k*) was found to be the highest for the Δt EMG-US (ICC: 0.75) and Δt US-FORCE (ICC: 0.7) while ICC values for the Δt EMG-MMG, Δt MMG-FORCE and Δt EMG-FORCE were found to be 0.48, 0.58, and 0.64, respectively. Results are shown in Table 2.

## Discussion

In our study, we synchronized ultrafast US with surface EMG, MMG and FORCE signals to detect time delays between EMG and MMG (Δt EMG-MMG), MMG and FORCE (Δt MMG-FORCE), EMG and US (Δt EMG-US), US and FORCE (Δt US-FORCE) and EMG and FORCE (Δt EMG-FORCE: EMD) during voluntary isometric contraction of the QF muscle. The high temporal resolution (4 kHz) of the US enabled to determine the onset of the fiber activation displayed as a signal of the first architectural change from the real anatomical depth of the RF muscle whilst preceding the surface EMG signal which was generated by voluntary isometric contraction of the QF muscle. In detection of the fiber activation, there was found higher repeatability using ultrafast US than the MMG. This method may provide more accurate information about the time course of the EC coupling and SEC when US probe is used to detect fiber activation during voluntary isometric contractions.

In our study, we attributed Δt EMG-US to the EC coupling during voluntary isometric contraction. Using ultrafast US, we were detected fiber activation after 19.1 ± 6.4 ms (SEM 1.15) preceding the onset of the EMG signal and this time delay was contributed 38.9% to the EMD. Using ultrafast US, other researchers detected fiber activation 6.05 ± 0.64 ms (52.5 ± 5.9% of EMD) in gastrocnemius (Nordez et al., 2009) and 4.43 ± 1.95 ms (56% of EMD) in biceps brachii (Hug et al., 2011a) after the time of the given electrostimulation, 2.2 ± 0.3 ms after EMG onset initiated by electrostimulation to the gastrocnemius muscle (Esposito et al., 2011a) and 21 ms in deep multifidus muscle after voluntary movement (Vasseljen et al., 2006). However, in our study, time delay Δt EMG-MMG also attributed to the EC coupling (Esposito et al., 2011a) was found to be 20.50 ± 4.73 ms, contributing 41.2% to the overall time delay (EMD) but with lower repeatibility (ICC: 0.48) than the Δt EMG-US (ICC: 0.75). On the other hand, using paired *t*-test, there was no statistically significant difference between EMG-MMG and EMG-US measurement methods (*p* > 0.05).

It is important to investigate time delays during voluntary muscle contractions rather than electrically induced contractions because there was reported that reduction in output from the motor cortex impairs EC coupling. (Goodall et al., 2009) The EC coupling names the process by which the depolarization at the T-system induces the release of Ca^2+^ from the cistern of sarcoplasmic reticulum. This process links the action potential to the force producing reactions (Gonzales and Rios, 2002). When EC coupling process is directly detected from the certain muscle depth using ultrafast US and tracking the onset of the fiber activation, it may enable to predict how EC coupling process is reflected out by the measure “ time” up to the point when SECs start to move. A number of the studies were used MMG signal to detect dimensional changes of the active muscle fibers but it was reported that small to moderate level of cross-talk is present between MMG signals from different locations when detected from the quadriceps femoris muscles during isometric contraction (Beck et al., 2010). This cross-talk was attributed to the tissues between muscle and MMG sensor acting as a low-pass filter (Beck et al., 2010). Subcutaneous fat may be thick enough to act as low-pass filter, reducing the gain factor of the MMG signal (Herda et al., 2010). To some extend, we can also attribute the MMG signal to the reflection of the muscle fiber activation, but tissues above the muscle acting as low-pass filter and low repeatability using MMG signal in our study (ICC: 0.48), may reduce its significance to be used as a detector of the fiber activation. Since quadriceps femoris muscle consists of 4 parts emerging into the only one tendon, adjacent muscles on the sides, vastus medialis and lateralis, and vastus intermedius below the RF, may also produce force tremor as a very important issue when muscles are simultaneously contracted. Indeed, in the present study, the reason for having a bigger time delay (even if not statistically significant) of the EMG-MMG (20.5 ± 2.22 ms) then the EMG-US (19.21 ± 3.43 ms) could be explained by the tissues above the muscle acting as low-pass filter and consequently increasing the time delay detected by MMG. Therefore, we strongly believe that US synchronized with surface EMG can provide more accurate informations about the timing of the EC coupling and confirm its relative contribution to the overall time delay. Further investigations with bigger sample size are needed to improve the accuracy and repeatability of the synchronized recording of the surface EMG and US during the voluntary muscle contractions.

In our study, we defined time delay Δt US-FORCE as a monitorization of the overall events after the onset of the fiber activation up to the time of the actual force production and attributed to the time course of the viscoelasticity of the SEC. Others defined this time also as an *elastic charge* time and attributed to the time interval between the onset of force production and joint motion (Winter and Brookes, 1991). In our study, it was found as 30.5 ± 8.8 ms, contributing 60.9% to the EMD, more than the EC coupling (38.9%) and with higher repeatability using US (ICC:0.7) then the MMG (ICC: 0.5). The time delay between MMG and FORCE output (Δt MMG-FORCE) was also attributed to the time after fiber activation up to the actual force production (Esposito et al., 2011a) and force transmission along the SEC of the MTU (Nordez et al., 2009). In electrically stimulated gastrocnemius muscle, the time delay between MMG and FORCE was found as 42.44 ± 3.07 ms. (Esposito et al., 2011a). Using high-rate US, the time delays corresponding to the aponeurosis and tendon of the gastrocnemius muscle were found as 2.37 and 3.22 ms, respectively, in total contributing 47.5 ± 6.0% to the EMD and lesser than EC coupling (Nordez et al., 2009). This time delay was also found as 4.43 ± 1.95 ms in biceps brachii muscle (Hug et al., 2011a). We have found that time delay corresponding to SEC was bigger than the EC coupling time and significantly different for both MMG and US trials (p < 0.05). Again, using the electrostimulation on the biceps brachii muscle, this time delay was increased from 7.9 ms to 19.6 ms as elbow was moved into deeper flexion while muscle-tendon length decreases. Author attributed this increase to the extend of slack and the shortening velocity of the fastest muscle fibers because muscle fibers should initially take up the slack and consequently to produce the movement (Sasaki et al., 2011). When measurements were performed in electrically stimulated muscles, none of these results could be compared with our results because we used voluntary isometric contraction as a trigger. On the other hand, the active effectiveness of the force transmission cannot be presented by electrically stimulated muscle without voluntary control. However, electrical stimulation delivers supramaximal stimulus which produces recruitment of different muscle fibers (Zhou et al., 1995) and consistent contractions (Hopkins et al., 2007). This might be reason why there was not found difference between time delays corresponding to the EC coupling and SEC when onset of the fascicle motion and tendon was detected by high rate US during electrostimulation of the biceps brachii muscle (Hug et al., 2011a). Measurements during active contraction may also reflect active stiffness characteristics of the SEC (Wilson et al., 1991). In our study, when MMG signal is accepted as an onset of the fiber activation, we can see that Δt MMG-FORCE is the biggest part (57.1%) of the EMD. When US onset is accepted as the onset of the fiber activation, Δt US-FORCE was contributed 60.9% to the EMD, also as a biggest part of the EMD. From our results, we can say that both viscous and elastic characteristics of the SECs preoccupy the biggest part of the EMD when tested during active muscle contraction. These time intervals, calculated between fiber activation and force output determines the time course required for the stretching of the tendon and aponeuroses (passive elements of the SEC) (Norman and Komi, 1979; Muraoka et al., 2004) during active muscle contraction. When Δt MMG-FORCE and Δt US-FORCE were compared with each other, no statistical significance was found, but higher repeatability was found using US than the MMG. It shows that ultrafast US could better reveal actual timing corresponding to the EC coupling and time required after fiber activation up to the force output. To the date, to our knowledge, this is the first study presenting duration of the viscoelasticity of the SECs during *active muscle contraction* using ultrafast US and comparing it with the MMG. Additionally, ultrafast US should be used in order to detect onset of the aponeurosis and tendon separately during active muscle contraction.

In our study, we defined the EMD (Δ*t* EMG-FORCE) as a time elapse between the onset of the surface EMG signal and actual force production when elicited by voluntary isometric contraction. It was found as 49.7 ± 6.99 ms with relatively low SEM and tendency to be repeatable (ICC: 0.64). Different methods have been used for measuring the EMD and this creates considerable difficulty when attempts are made to compare data. However, we could say that our results were not differed in huge extend from others which were reported as 39.6 ms (Winter and Brookes, 1991), 38.7 ms (Zhou et al., 1995), 57.2 ms (Howatson et al., 2009), 37.8–56.5 ms (Zhou, 1996) and 40–60 ms (Hug et al., 2011b). There are many reasons affecting the EMD such as recruitment of the fiber type depending on the contraction velocity, inhomogenous muscle activation, muscle and tendon stiffness, rate of force production, gender, temperature, fatigue and hormonal characteristics (Winter and Brookes, 1991; Yavuz et al., 2010; Hug et al., 2011b; Cè et al., 2013; Earp et al., 2014). Muscle and tendon stiffness are very important factors, mostly changed after orthopedic surgery caused by the scar tissue development, inproper body ergonomics, and malalignment. It was shown that EMD of the hamstring muscle significantly increased after harvesting hamstring tendon what can affect the knee safety and performance (Ristanis et al., 2009). On the other hand, it was also reported that EMD of the vastus medialis oblique muscle was longer then vastus lateralis muscle in patients with patellofemoral pain syndrome (Chen et al., 2012). Thus, from the previous reports and present study, we suggest that monitoring of the EMD could be useful for both diagnostic and rehabilitation purposes. Even more important if EMD is measured during the active muscle contraction because ligament afferents play an important role in the regulation of the functional articular stability, continuous control of muscle activities and programming the muscle stiffness (Mora et al., 2003). Thus, synchronization of the surface EMG with ultrafast US and force sensors should be increasingly utilized to detect differences caused by disabilities in order to create more effective rehabilitation programs. The repeatability of the EMD during active muscle contractions should be improved in further investigations.

There are some limitations which are needed to be mentioned when interpreting results to our study. Analysing the signals displayed by active contraction might be challenging. Even if we had been given verbal instructions to the subject to keep itself relaxed before exerting contraction, sitting on the chair of the dynamometer for the certain period and many sensors attached on the leg as well as increased attention might be reasons for having discomfort which may cause increase of the baseline noise of the signal. In some contractions, baseline noise was such small that onset could be detected earlier but this was not case in most contractions in our study. Therefore, we set the threshold of the three SDs of the mean baseline noise and used it consistently during our signal analysis. Other important consideration should be given to our interpretation of the force output because force is exerted as a sum of all parts of the quadriceps femoris muscle. We used time delays to determine EMD but future studies should be focused on the separate detection of the time delays from each part of the QF muscle and to find out their relative contributions to the EMD during active muscle contractions.

## Conclusion

In conclusion, using ultrafast US to detect fiber activation and synchronizing it with the surface EMG and FORCE sensors, revealed more reliable results than using the MMG sensor. With ultrafast US, we can detect onset from the certain depth of the contracting muscle excluding the tissues above the muscle acting as low-pass filters which can lead to inaccurate time detection of the onset of the contracting muscle fibers. Monitoring the EMD and its components which are time course of EC coupling and SECs during active muscle contraction, could better unveil spinal and supraspinal pathologies and their pathologic reflection on the peripheral nervous system and muscle dynamics. Thus, synchronization of the surface EMG with ultrafast US and force sensors should be increasingly utilized to detect differences caused by disabilities in order to create more effective rehabilitation programs. Further investigations are needed to improve the accuracy and repeatability of the synchronized recording method during the voluntary muscle contractions.

### Conflict of interest statement

The authors declare that the research was conducted in the absence of any commercial or financial relationships that could be construed as a potential conflict of interest.

## Abbreviations

ACL: Anterior cruciate ligament
ANOVA: Analysis of variance
CC: Contractile component
CV: Coefficient of variation
EC: Excitation-contraction
EMG: Electromyography
EMD: Electromechanical delay
FORCE: Force output
MMG: Mechanomyography
ICC: Intraclas correlation coefficient
MTU: Muscle-tendon unit
RF: Rectus femoris
RMS: Root mean square
ROI: Region of interest
SEC: Series elastic component
US: Ultrasonography.
